# Recent Methods for Evaluating Crop Water Stress Using AI Techniques: A Review

**DOI:** 10.3390/s24196313

**Published:** 2024-09-29

**Authors:** Soo Been Cho, Hidayat Mohamad Soleh, Ji Won Choi, Woon-Ha Hwang, Hoonsoo Lee, Young-Son Cho, Byoung-Kwan Cho, Moon S. Kim, Insuck Baek, Geonwoo Kim

**Affiliations:** 1 Department of Biosystems Engineering, College of Agricultural and Life Sciences, Gyeongsang National University, 501, Jinju-daero, Jinju-si 52828, Gyeongsangnam-do, Republic of Korea; bpagoe@gmail.com (S.B.C.); hdytsoleh@gmail.com (H.M.S.); jiwonbb626@gnu.ac.kr (J.W.C.); 2 Division of Crop Production and Physiology, National Institute of Crop Science, Rural Development Administration, 100, Nongsaengmyeong-ro, Iseo-myeon, Wanju-gun 55365, Jeonbuk-do, Republic of Korea; hwangwh@korea.kr; 3 Department of Biosystems Engineering, College of Agriculture, Life and Environment Sciences, Chungbuk National University, 1 Chungdae-ro, Seowon-gu, Cheongju-si 28644, Chungbuk-do, Republic of Korea; 4 Department of Smart Agro-Industry, College of Life Science, Gyeongsang National University, Dongjin-ro 33, Jinju-si 52725, Gyeongsangnam-do, Republic of Korea; protaetiacho@gnu.ac.kr; 5 Department of Biosystems Machinery Engineering, Chungnam National University, Daejeon 34134, Republic of Korea; chobk@cnu.ac.kr; 6 Environmental Microbial and Food Safety Laboratory, Agricultural Research Service, Department of Agriculture, Powder Mill Road, BARC-East, Bldg 303, Beltsville, MD 20705, USA; moon.kim@usda.gov (M.S.K.); insuck.baek@usda.gov (I.B.); 7 Institute of Agriculture and Life Sciences, Gyeongsang National University, 501, Jinju-daero, Jinju-si 52828, Gyeongsangnam-do, Republic of Korea

**Keywords:** crops, water stress, machine learning, deep learning, artificial intelligence (AI)

## Abstract

This study systematically reviews the integration of artificial intelligence (AI) and remote sensing technologies to address the issue of crop water stress caused by rising global temperatures and climate change; in particular, it evaluates the effectiveness of various non-destructive remote sensing platforms (RGB, thermal imaging, and hyperspectral imaging) and AI techniques (machine learning, deep learning, ensemble methods, GAN, and XAI) in monitoring and predicting crop water stress. The analysis focuses on variability in precipitation due to climate change and explores how these technologies can be strategically combined under data-limited conditions to enhance agricultural productivity. Furthermore, this study is expected to contribute to improving sustainable agricultural practices and mitigating the negative impacts of climate change on crop yield and quality.

## 1. Introduction

Despite international efforts to reduce greenhouse gas emissions over the past several decades, the global surface temperature has increased by approximately 2.45 °C compared to the 19th century. This global warming has triggered sudden natural disasters worldwide, including heat waves, cold waves, heavy rain, droughts, and floods [1]. Additionally, these conditions can significantly affect agricultural ecosystems, especially climate conditions [2]. Consequently, the impact of climate change has contributed to declines in the yield and quality of agricultural products [3]. Crop damage caused by climate change has been reported in several countries, including Thailand, India, China, and the United States. Hence, various studies have been conducted in an effort to reduce the impacts of climate change over recent years [4,5]. These impacts have been highlighted not only in popular media, such as the news, but also in a lot of relevant research [5,6,7,8].

Currently, the agricultural industry consumes 80–90% of the world’s freshwater resources [9], and the intensity and frequency of precipitation caused by climate change are expected to increase the demand for irrigation water. Under these circumstances, one of the key elements of sustainable agricultural applications is to monitor the crop water stress levels [10,11]. Water stress evaluation can help to control the amount of water used and prevent excessive water consumption, which significantly impacts crops’ yield and quality.

Traditional methods for evaluating crop water stress involve measuring the soil moisture content and analyzing meteorological variables and various physiological parameters, such as water potential and stomatal conductivity [12,13]. Although these methods can provide direct information about the crop water stress level, the traditional methods are time-consuming, laborious, and destructive [14]. These are significant drawbacks for the evaluation of water stress levels in field crops. Moreover, using traditional methods would make it difficult to cover large areas [15,16].

Non-destructive and rapid crop water stress monitoring technologies have been developed to overcome these limitations. Remote sensing coupled with various optical sensors (RGB, thermal, multispectral, and hyperspectral imagery) is used, and these platforms are also applied to satellites, aircraft, drones, and handheld devices for the rapid collection of digital data. These digital data can be analyzed using artificial intelligence (AI) techniques to assess the chemical and physical properties of crops [17,18,19]. Remote sensing technologies have been applied for crop classification [20], yield prediction [21], the detection and management of diseases and pests [22], and crop water stress detection [23]. These technologies generate large datasets, including open-source satellite data from platforms such as Google Earth Engine [24,25]. To transform the collected data into meaningful information, various preprocessing steps are required. However, manually analyzing such vast amounts of data is time-consuming. Hence, AI technology is employed to address this challenge.

The use of artificial intelligence (AI) for crop water stress analysis began in earnest in the mid-1970s. For instance, Millard et al. [26] conducted a study in April 1976, measuring crop temperatures using infrared scanners and IR photography from both aircraft and ground platforms in wheat fields subjected to various water stress levels. Since then, AI technologies have played important roles in predicting optimal irrigation timing and quantity, reducing water waste, and increasing crop yields [27]. These technologies have improved the efficiency of irrigation systems and water management. In particular, AI-based drones and satellite systems enable precise irrigation monitoring for crop health, soil moisture levels, and water usage across large areas [28].

To date, AI has been widely used in crop identification [16,29], disease detection [30], and yield prediction [31]. Specifically, for crop water stress assessment, machine learning and deep learning algorithms are primarily employed. Several techniques, such as Random Forest (RF), Support Vector Machine (SVM), and Convolutional Neural Network (CNN) architectures, are intensively used, and generative AI models such as Generative Adversarial Network (GAN) models have recently been applied [32].

Accordingly, this study comprehensively reviewed the trends of various sensing platforms utilizing machine learning and deep learning techniques for crop water stress analysis over the past decade. The primary objective of this study was to present the AI techniques used in crop water stress analysis and to evaluate the related technical methods used. Specifically, we focused on collecting and analyzing AI techniques utilized in remote sensing technologies. While most previous studies have concentrated on thermal imaging, multispectral/hyperspectral sensors [13,33], or water stress studies on specific crops [34,35,36], excluding RGB, this study analyzed not only these sensing technologies but also AI techniques for evaluating water stress in a variety of field crops.

This study provides an in-depth analysis of how AI technologies can contribute to managing crop water stress and promoting efficient water resource utilization; it also aims to offer solutions for agricultural challenges caused by extreme climate conditions. In addition, optimal approaches to maximize the applicability and effectiveness of AI-based technologies in agriculture are suggested. The findings from this analysis are expected to contribute to strategies ensuring the sustainability of agriculture in the future and to further advance the application of AI technologies in the agricultural sector.

## 2. Materials and Methods

To investigate the latest trends in remote sensing and artificial intelligence techniques for evaluating crop water stress in the context of climate change, a systematic process of literature collection and screening was conducted. The primary databases selected for this purpose were Google Scholar, Scopus, Web of Science, and ScienceDirect. Google Scholar provides a broad range of resources across various academic fields, while Scopus offers citation analysis, allowing for the evaluation of research’s impact and quality. Web of Science is particularly strong in citation counts and impact factor analysis, while ScienceDirect offers access to the latest research in science, technology, and medicine. These databases are well-suited for a comprehensive review of studies related to agriculture, remote sensing, and artificial intelligence.

For the literature search, keywords such as “Machine learning”, “Deep learning”, “Water stress”, “Crop”, “Remote sensing”, and “Climate change” were used to target journal and conference papers published over the past decade (2013–2024). The search terms were restricted to appear in the “title”, “abstract”, or “keywords” sections of the papers. The search scope was limited to “journals”, and the document types included “research articles”, “reviews”, and “articles in press”. Additionally, the search was restricted to papers published in English.

Through this process, approximately 130 papers were collected, and additional literature was gathered using supplementary keywords such as “RGB”, “Thermal imaging”, “CWSI”, and “Hyperspectral”. Given the practicality of hyperspectral techniques in evaluating crop water stress, multispectral technology was also investigated. Table 1 provides a summary of the search strings used for each database.

During the literature collection process, basic keywords such as “Machine learning”, “Deep learning”, “Water stress”, “Crop”, “Remote sensing”, and “Climate change” were initially used. However, many irrelevant materials were retrieved, and some necessary studies were omitted. To address this issue, the search was refined in over 20 iterations by adding or removing keywords to identify the optimal search terms. Additional key terms, including “RGB”, “Thermal”, and “Hyperspectral”, were also employed. As a result, approximately 95 papers were collected, and the literature was categorized according to remote sensing techniques in order to avoid duplication and clarify the application cases of each technology.

- Examples of crop water stress assessment using RGB imaging;
- Examples of crop water stress assessment using thermal imaging;
- Examples of crop water stress assessment using CWSI;
- Examples of crop water stress assessment using hyperspectral imaging.

The selected articles were re-filtered based on their relevance to crop water stress assessment, using the following exclusion criteria to strengthen the focus and relevance of the research: First, review papers were excluded. Although review papers provide comprehensive analyses of existing studies, they do not present specific experimental data, which were necessary for this research. Second, studies employing destructive methods were also excluded. Destructive methods are not suitable for maintaining crops in actual agricultural environments, making non-destructive techniques more favorable. Third, studies that did not utilize AI-based approaches were excluded. AI methods are critical for enhancing the precision of crop water stress assessments, and they reflect the latest advancements in technology. Lastly, studies that focused on crops that were not subjected to water stress were excluded, as the primary goal of this research was to evaluate crop water stress, placing such studies outside our scope.

- Review articles;
- Assessment of crop water stress with destructive methods;
- No AI learning;
- Crops not under water stress.

**Figure 1:**
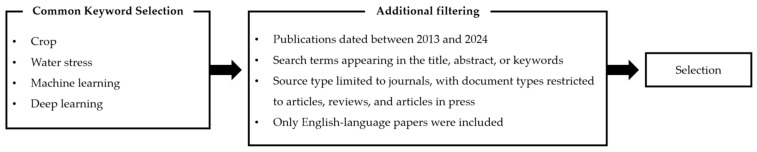
Paper selection criteria.

**Figure 2:**
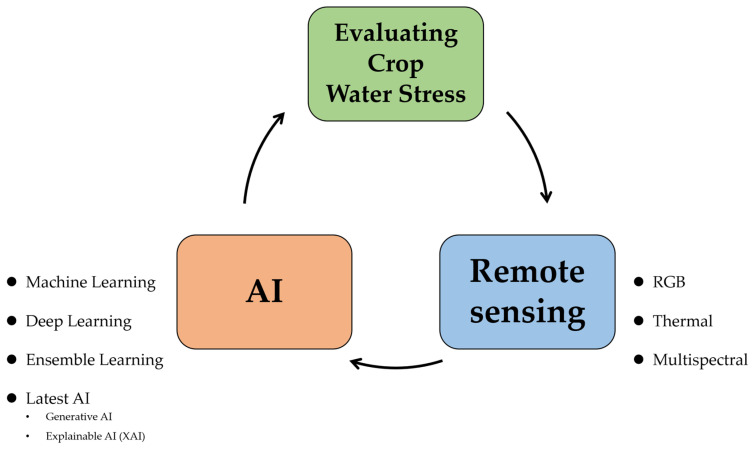
Paper flowchart.

In the end, 46 articles were selected. The selected articles were published by Elsevier, Springer, MDPI, IEEE, etc., and were selected based on the publishers that are commonly selected for literature reviews. The keywords and additional filtering choices for the literature selection are shown in Figure 1. The flow of the paper is shown in Figure 2.

## 3. Remote Sensing

**Figure 3:**
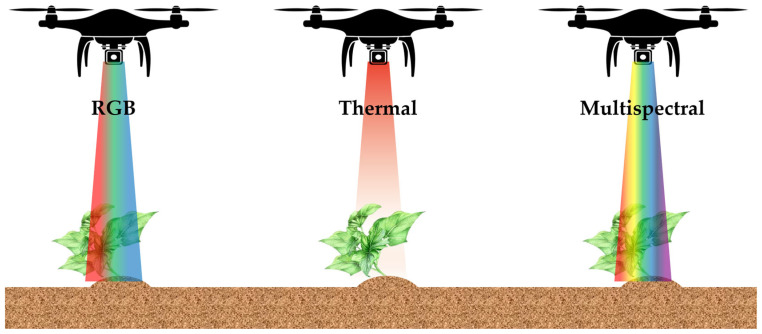
RGB, thermal, and multispectral remote sensing. RGB images capture the color and growth status of crops through visible light, while thermal imaging sensors detect temperature changes in crops to identify water stress or diseases. Multispectral imaging utilizes multiple wavelengths of light to analyze the physiological responses and health conditions of crops in greater detail.

Methods for measuring water stress in crops are based on the interaction between the crop and soil. In general, measuring crops’ water status and soil moisture content directly in the field is laborious, time-consuming, and destructive [37,38,39], so there is a need for a time-saving, accurate, easy, and non-destructive method to detect crops’ water status in order to curb yield and economic losses early [40]. Recent research to assess crop water stress has been conducted by using remote sensing data as an alternative to traditional measurement methods. The advantage of using remote sensing is the provision of information on the spatial and temporal variability of crops, allowing for more comprehensive analysis and forecasting [41,42,43,44,45,46,47]. A brief description of the remote sensing techniques used to assess water stress in crops is provided below (Figure 3).

### 3.1. RGB Imaging

Using the literature review methodology described above, a total of five papers were selected in which RGB imaging technology was used for crop water stress assessment. RGB imaging is the simplest remote sensing technique for crop detection, based on silicon sensors that are sensitive to the visible-light band (400–750 nm) and capable of two-dimensional imaging. Typically, the raw data of an image are represented as a matrix of intensity values corresponding to photon fluxes in the red (~600 nm), green (~550 nm), and blue (~450 nm) spectral bands of visible light. RGB images are widely used in crop science because of their low cost and ease of operation and maintenance [48]. Therefore, a variety of deep learning and machine learning techniques have been used to assess crop water stress [49,50,51]. The following Table 2 summarizes the use of RGB imaging to assess moisture stress in crops; it is organized by crop type, best-performing model, methodologies used, paper objectives, author, publisher, country, and year.

Monitoring crops’ water content using RGB imaging requires preset lighting conditions as well as specific leaf orientation for the camera. This limits the applicability of RGB imaging for assessing moisture content in the field [48]. Therefore, the use of RGB imaging for crop water stress assessment is considered to be limited and is mainly used in conjunction with thermal imaging techniques [57,58].

### 3.2. Thermal Imaging

Through the literature review methodology described above, a total of 13 papers were selected in which thermal imaging techniques were used for crop water stress assessment. High-resolution thermal imaging cameras have a spectral range of 3–14 μm, with the most commonly used wavelengths being 3–5 μm or 7–14 μm [48]. Thermal imaging cameras are relatively more expensive than simple-to-operate RGB cameras, which also have limited features when used for crop water assessment. However, thermal images perform better than RGB images in analyzing crop moisture stress, because thermal images are more reliable and sensitive to changes in crop moisture content due to their higher penetration compared to RGB wavelengths. Therefore, thermal imaging is a more suitable imaging technology for crop moisture stress analysis than RGB imaging [59,60]. The following Table 3 summarizes the use of thermal imaging to assess water stress in crops, and it is organized by crop type, best-performing model, methodologies used, paper objectives, author, publisher, country, and year of publication.

### 3.3. CWSI

Using the literature review methodology described above, a total of 10 articles were selected in which CWSI technology was used to assess crop moisture stress. Moisture stress is one of the most influential factors contributing to crop yield losses. Water deficit during critical stages of growth, such as during vegetative growth, flowering, or fruit development, can lead to significant yield losses [72,73]. Previous studies have used canopy temperature as an efficient way to rapidly and non-destructively monitor crops’ responses to water stress [74,75], This revealed that canopy temperature provides important clues to changes in the water status and yield of crops under stress and non-stress conditions during drought periods [76,77]. The Crop Water Stress Index (CWSI), based on canopy–air temperature difference and vapor pressure deficit (VPD), has been developed and is a promising tool for assessing water stress in crops [74]. The expression for CWSI is as follows:

$$CWSI=\frac{T_{l}-T_{wet}}{T_{dry}-T_{wet}}$$

where $T_{l}$ is the temperature of the leaf; $T_{wet}$ is the lower bound of the canopy temperature, corresponding to a well-watered leaf with fully open stomata; and $T_{dry}$ is the upper bound of the canopy temperature, corresponding to a leaf with fully closed stomata, i.e., a non-permeable leaf [78]. Previous research has shown that the CWSI takes less time to detect water stress at the farm level because it can measure water stress remotely; hence, this method was the most commonly used indicator for assessing water stress in crops [79,80,81,82]. However, the CWSI has not been adopted in several applications, due to the following reasons [78]: (i) Temperatures from the associated crop canopy, general leaf population, and soil backgrounds, which are mixed when measured by handheld or high-altitude airborne radiometers. (ii) The normalization of the CWSI is much more complex when atmospheric conditions change than using VPDs only [83,84,85]. However, we believe that widespread use of the CWSI could be a viable option if high-resolution canopy temperature can be accurately monitored [79]. The following Table 4 summarizes the use of the CWSI to assess water stress in crops, and it is organized by crop type, best-performing model, the methodologies used, paper objectives, author, publisher, country, and year.

### 3.4. Hyperspectral Imaging

Through the literature review methodology described above, a total of 18 articles were selected that used multispectral and hyperspectral imaging techniques for crop water stress assessment. The application of imaging spectroscopy for crop phenotyping originated from studies on the remote sensing of vegetation [48]. Imaging spectroscopy is a technique for detecting and classifying objects by measuring the light reflectance of finely divided wavelengths in the optical part of the electromagnetic spectrum [96]. However, multispectral satellite remote sensing cannot effectively detect early signs of stress in crops (e.g., nutrient deficiencies, crop diseases) in a timely manner, as the accuracy of the retrieved variables is often limited due to limitations in spectral resolution [97]. This has led to the need for remote sensing instruments and sensors with high spectral and spatial resolution [98]. The use and development of hyperspectral imaging have been crucial to eliminating those problems, providing hundreds of bands from which to obtain a more detailed spectral response of the target feature than multispectral imaging [20]. The following Table 5 summarizes the use of hyperspectral imaging to assess water stress in crops, and it is organized by crop type, best-performing model, the methodologies used, paper objectives, author, publisher, country, and year.

## 4. Artificial Intelligence

### 4.1. Machine Learning

Machine learning is an evolving field of computational algorithms that are designed to imitate human intelligence by learning from their surroundings. This field has a major role to play in the new era of big data [117]. Machine learning uses algorithms that learn from data, allowing computers to teach themselves information from data to solve problems. These learning algorithms are used in many fields, including image processing, prediction, analytics, and more [118]; they are broadly divided into supervised learning, unsupervised learning, and reinforcement learning.

1. Supervised learning is a method of using pairs of input data and corresponding output values to learn a function that allows the system to predict the output for new inputs [119].
2. Unsupervised learning is a method of classifying patterns among data by uncovering the hidden structure of input data without an output [120].
3. Reinforcement learning is a subfield of machine learning in which software agents learn behaviors that maximize their cumulative reward in the environment [121].
4. Reinforcement learning (RL) offers distinct advantages for real-time decision-making and automation in agriculture; its capacity to continuously learn and adapt through interactions with the environment makes it especially effective for dynamic and changing agricultural conditions. Although the use of RL in crop water stress research is currently limited [122], its potential to greatly enhance adaptive management and optimize irrigation strategies suggests that further exploration and experimentation are worthwhile [123,124,125].

The selection of the algorithm approach depends on the type of problem to be solved, the number of variables involved, and the type of model that best fits the data, among other factors [118]. In particular, SVM and PLS algorithms have been effectively used to analyze remote sensing data as models for crop water stress assessment. The following Table 6 is a summary of the use of machine learning for crop water stress analysis in the above research cases.

#### 4.1.1. Support Vector Machines

Support Vector Machines were introduced by Vapnik in 1995 and are classification models based on statistical learning theory that can be applied to both classification and regression problems [126]. Although SVMs were developed in the late 1970s, they started to gain popularity in the field of remote sensing in 2003 [127]. SVMs primarily aim to find the hyperplane that maximizes the margin between two classes [128]. When data are linearly separable, SVMs separate the two classes using the hyperplane that achieves the widest margin. However, in cases where the data are not linearly separable, SVMs use kernel functions to map the data into a higher-dimensional feature space, where an optimal hyperplane is found. SVMs can utilize various kernel functions (e.g., linear kernel, polynomial kernel, and Gaussian kernel) to map nonlinear data into higher dimensions, and choosing an appropriate kernel function significantly affects their classification performance [129].

**Figure 4:**
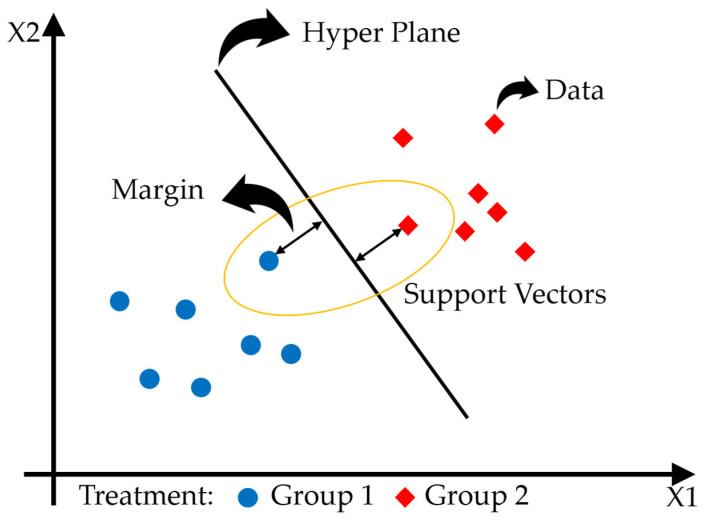
The structure of the SVM is shown, with X1 and X2 as input features. The yellow circle highlights the support vectors, which help to define the margin and the hyperplane separating Group 1 and Group 2.

In this process, support vectors are the most critical data points that contribute to defining the hyperplane separating the two classes. The remaining data points do not influence the position of the hyperplane, which is one reason SVMs can achieve high classification accuracy even with a small amount of data [130]. Additionally, SVMs are known to be robust against overfitting, as they strike a balance between performance on the training data and the ability to generalize to new data [127]. The following Figure 4 shows the structure of the SVM.

Such SVMs are utilized in various fields, including medicine [131,132], statistics [133,134], and text analysis [135,136]. In the agricultural sector, SVMs are particularly applied in crop prediction and classification [137,138], as well as in yield forecasting [139,140].

#### 4.1.2. Partial Least Squares Regression

Partial Least Squares Regression was introduced by H. Wold in 1975 [141]. Developed to handle large datasets, PLS combines path analysis, principal component analysis, and regression analysis [142,143], integrating dimensionality reduction with parameter estimation. PLS iteratively applies simple bivariate regression (least squares) between columns or rows of matrices in order to estimate covariates for each model. The process begins by generating latent factor variables from the independent variable data (X matrix), which are then used to model the relationship with the dependent variable (Y). In a PLS model, the contribution of each variable is evaluated through standardized model coefficients, which represent the relationships between variables. A larger positive coefficient indicates that the independent variable has a stronger positive influence on the dependent variable [143,144]. This method is particularly useful for modeling complex relationships involving multiple variables, making it suitable for exploratory research or studies where complex causal relationships are not yet fully understood [145,146].

**Figure 5:**
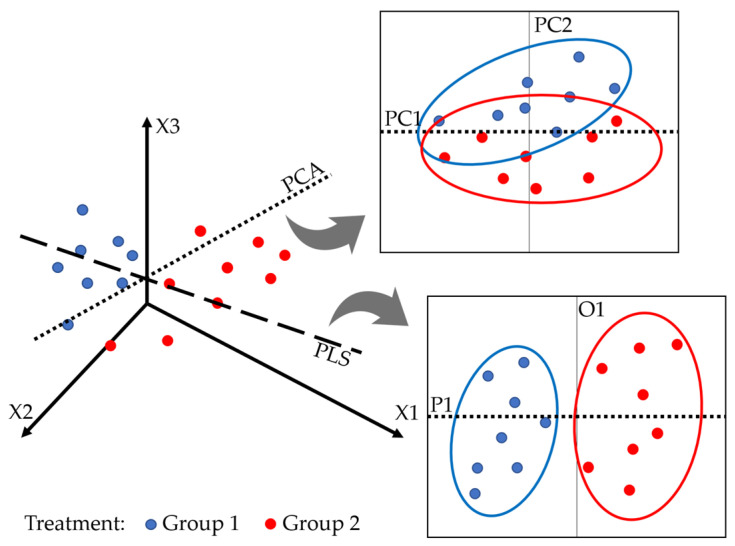
X1, X2, and X3 are the original features. PC1 and PC2 are principal components from PCA, while P1 and O1 are components from PLS.

PLS is often confused with principal component analysis (PCA). PCA transforms the original set of variables into principal components (PCs), where the first principal component explains most of the data’s variance, and subsequent components account for progressively less variance. Unlike PLS, which models the relationship between independent variables (X) and dependent variables (Y), PCA focuses on explaining the variance within the independent variables (X) alone, without considering their relationship to the dependent variable (Y) [147,148]. The following Figure 5 shows the structure of the PLS.

PLS is applied in various fields, including technology adoption analysis [149], leisure studies [150], linguistics and education [151], and marketing [152]. In the agricultural sector, PLS is primarily used for analyzing the behavior of agricultural practitioners [153,154,155].

### 4.2. Deep Learning

Deep learning is an extension of classical machine learning, using a variety of functions to add more “depth” to models and represent data in a hierarchical way with multiple levels of abstraction [156,157]. One of the benefits of deep learning is feature learning; it automatically extracts features from raw data, with higher-level features in a hierarchy formed by combinations of lower-level features [158]. Deep learning also uses more complex models than machine learning, allowing for massively parallel processing. These deep learning models excel at classification and prediction due to their hierarchical structure and large learning capacity, and they are flexible enough to adapt to diverse and complex data analyses [159]. Deep learning has been applied to a wide range of fields, including automatic speech recognition, image recognition, natural language processing, drug discovery, and bioinformatics [160]. Deep learning is a relatively new technology, especially in agriculture; however, many researchers have tried to implement it in several applications, such as disease detection and identification, fruit and object classification, and many more [161]. In particular, ANN, CNN, and RNN algorithms have been effectively used to analyze remote sensing data as models for crop water stress assessment. The following Table 7 is a summary of the use of deep learning for crop water stress analysis in the above case.

#### 4.2.1. Artificial Neural Networks

**Figure 6:**
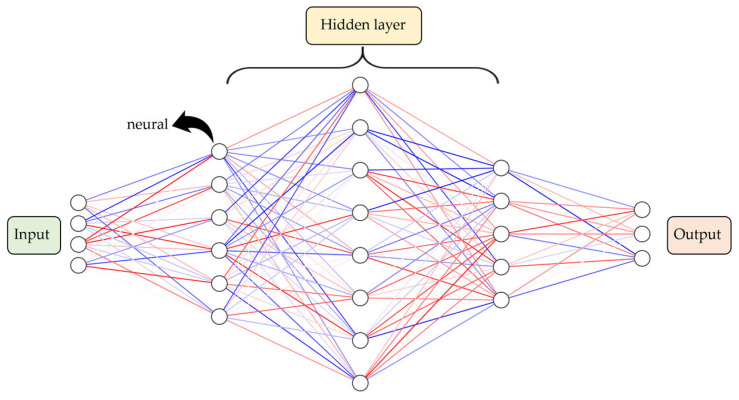
Artificial Neural Network structure. This figure depicts the structure of an Artificial Neural Network (ANN) composed of an input layer, hidden layers, and an output layer; it shows how each node is interconnected to process data and derive output values.

The concept of an ANN, introduced by W.S. McCulloch and W. Pitts, is a mathematical representation of the neurons in the human brain, designed to simulate the way in which the brain processes information [162]. ANNs began to be widely used in research with the introduction of the backpropagation (BP) training algorithm for feedforward neural networks in 1986 [163]. ANNs are biologically inspired computational models composed of hundreds of single-unit artificial neurons that are trained to adjust their parameters in order to produce outputs similar to those of known datasets [164]. By learning from historical data, once sufficiently trained, ANNs can adapt to recurring changes and detect patterns in complex data [165,166]. One of the most prominent types of ANN is the Multilayer Perceptron (MLP) neural network, which consists of an input layer, an output layer, and one or more hidden layers in between, with each layer containing multiple artificial neurons. These neurons receive input signals, apply weights to calculate a weighted average, and generate outputs through an activation function [167]. The following Figure 6 shows the structure of the ANN.

ANNs can perform regression analysis on highly nonlinear problems and are applied to find nonlinear relationships between input and output datasets [168]; they are mainly utilized for classification and recognition using multispectral information [169]. In agriculture, ANN models have been used in crop development modeling [170], crop yield prediction [171,172], evapotranspiration estimation [173], and crop water stress assessment [174,175,176].

#### 4.2.2. Convolutional Neural Networks

**Figure 7:**
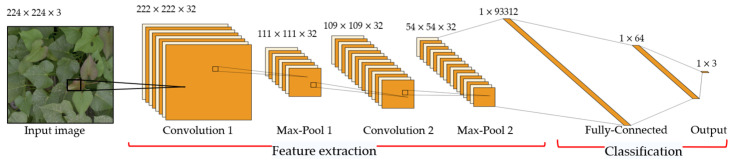
The input image (224 × 224 × 3) passes through convolution, max-pooling layers for feature extraction, and fully connected layers for classification. Black squares indicate focused regions at each layer.

In 1980, K. Fukushima proposed the neocognitron, which can be considered to be the predecessor of CNNs [176]. In 1990, LeCun et al. [177] published a seminal paper that established the modern framework for CNNs, and since the early 2000s, ConvNets have had great success in detecting, segmenting, and recognizing objects and regions in images [158]. The basic building blocks of a CNN consist of three types: convolutional, pooled, and fully connected layers [178]. The convolutional layer detects local connections between features from previous layers, and the pooling layer merges semantically similar features into one [158]. The following Figure 7 shows the structure of the CNN.

CNNs can automatically learn important features from images and find hidden patterns. When learning more data, this system can be more advanced at finding deep features in images [179]. In agriculture, CNN models are being used for crop mapping [180], crop disease diagnosis [181,182,183,184], weed and crop recognition [185,186], yield prediction [187], and crop water stress detection [57].

### 4.3. Ensemble Learning

One of the earliest examples of ensemble learning is the work of Dasarathy and Sheela in 1979, which introduced the idea of partitioning feature space using multiple classifiers [188]. Since then, there has been an explosion of research on ensemble learning, with the main methods being bagging, boosting, and stacking [189,190]. Ensemble learning is a method that combines multiple base learners to make predictions on new inputs. Bayesian learners consist of a variety of machine learning algorithms, such as decision trees, neural networks, and linear regression, which take labeled data as inputs and create a predictive model. This method allows for predictions on new, unlabeled data [191]. Such ensemble learning can reduce the risk of overfitting owing to a variety of base models, and by combining the results of different classification algorithms, it can reduce generalization error without increasing the variance of the model [192]. In addition, traditional ensemble learning has been applied to a variety of fields by incorporating basic machine learning models [193,194]. However, in recent years, there have been many attempts to apply deep learning to ensemble learning [195,196]. Ensemble learning has a wide range of applications, including fake news detection [197], web-based attack detection [198], battery health estimation [199], dissolved oxygen prediction [200], and short-term electricity load prediction. In the agricultural field, ensemble learning has been used for growth diagnostics [201], yield prediction [202], pest classification [203], disease recognition [204], and crop classification [205]. XGBoost and RF algorithms have been effectively used to analyze remote sensing data to assess crop water stress. The following Table 8 are examples of how ensemble learning algorithms are used to analyze crop water stress in the above cases.

#### 4.3.1. Extreme Gradient Boosting

**Figure 8:**
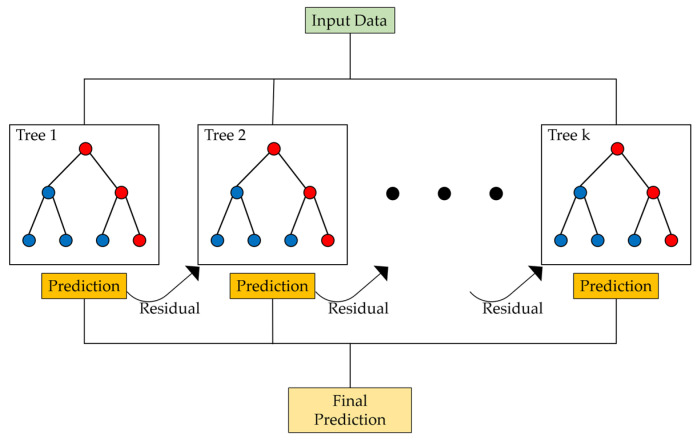
Boosting algorithm structure (red circles: incorrect prediction and blue circles: correct prediction).

Extreme Gradient Boosting is an algorithm based on the boosting tree model, introduced by Tianqi Chen and Carlos Guestrin in 2014, which is optimized for decision and regression trees [206]. The gradient boosting algorithm was developed for its very high predictive power; however, it has the disadvantage that it requires a lot of training time because one decision tree must be created at a time to minimize the error of the previous trees in the model. XGBoost was created to eliminate this drawback [207]. XGBoost primarily utilizes gradient-boosted decision trees, emphasizing speed and performance. Boosting is an ensemble method that adds new models to correct the errors of existing models. XGBoost generates a new model to predict the residuals (errors) left by the previous models and then adds it to the existing models to improve the final prediction. When adding new models, the algorithm uses gradient descent to minimize errors [208]. The following Figure 8 shows the structure of the Boosting algorithm. It illustrates the structure of the boosting algorithm, where multiple decision trees are sequentially trained.

XGBoost is very effective at reducing computation time and making optimal use of memory resources [209]. In the agricultural sector, XGBoost is being used in various fields, such as yield prediction [210,211,212], evapotranspiration prediction [213], crop forecasting [214], etc.

#### 4.3.2. Random Forest

**Figure 9:**
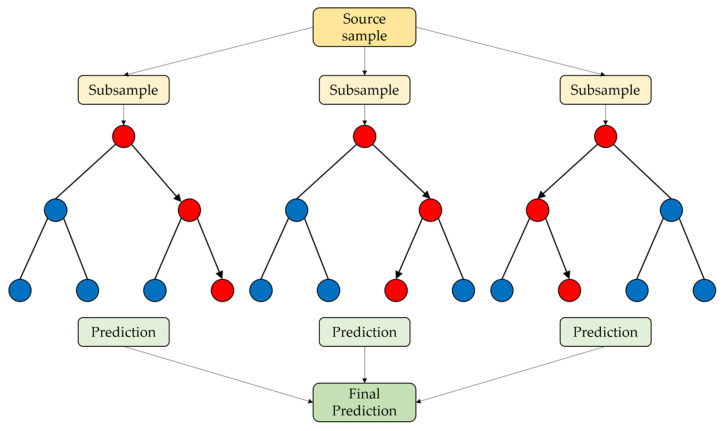
Bagging algorithm structure (red circles: incorrect prediction and blue circles: correct prediction).

Random Forest was introduced by L. Breiman in 2001 [215]. RF is an ensemble machine learning algorithm that uses a subset of features and bootstrap samples to create regression trees [216]. The essential components of this ensemble are predictors with a tree structure, and each tree is generated by introducing randomness into the process, which is why this procedure is referred to as a “Random Forest” [217]. Random Forest is an algorithm that generates multiple decision trees through randomization and then trains them using bootstrap sampling; for classification tasks, it derives the final prediction through voting, while for regression tasks, it averages the predictions. Each tree is created by randomly selecting predictors and determining the optimal splits, and “out-of-bag” (OOB) data are used to evaluate the model’s performance [218]. The following Figure 9 shows the structure of the Bagging algorithm. Figure 9 demonstrates the process of the bagging algorithm, where multiple samples are drawn, and each decision tree is trained separately. The predictions from each tree are then aggregated to produce the final prediction.

The RF algorithm performs efficiently on large databases and can handle thousands of input variables without overfitting, achieving fast and high prediction accuracy [215]. In agriculture, RF is used in a variety of applications, including crop classification [219,220], yield prediction [221], and crop forecasting [222].

## 5. Case Analysis

In this section, we systematically organize the collected cases according to their respective remote sensing and AI techniques. Subsequently, we present the results of specific studies that evaluate and compare the performance of each technique. Through this analysis, we aim to provide a clearer understanding of the strengths and limitations of each technique. The cases have been organized based on the most frequently used AI techniques and have been analyzed in detail, considering aspects such as study area, study period, data acquisition methods, number of data, and accuracy. The specific applications are as follows:

### 5.1. SVM

Azimi et al. [52] proposed a method for identifying water stress in chickpeas based on RGB images. The SIFT and HOG feature extraction techniques were employed. For analysis, KNN, decision trees, Naive Bayes, and SVM were used, with SVM achieving the highest accuracy of 73%.

Mohite et al. [107] proposed a method for detecting water stress in maize crops using drone-based hyperspectral imagery. The hyperspectral data from the influential wavelength bands were utilized for water stress detection. SVM and RF were used for analysis, with SVM demonstrating the highest performance in the 670–780 nm wavelength range.

Sankararao et al. [108] proposed a method for detecting water stress in pearl millet crops using drone-based hyperspectral imagery. Hyperspectral data were processed with various machine-learning-based feature selection techniques to extract wavelength bands sensitive to water stress. SVM and RF were used for analysis, with SVM achieving an accuracy of 95.38%. For early stress detection, the method achieved an accuracy of 80.76%.

Zhuang et al. [113] proposed a method combining continuous wavelet transform and machine learning techniques to predict the water status of winter wheat. The wavelet transform decomposed data into frequency components at various scales, enabling simultaneous analysis of time and frequency information. The resulting multi-wavelength features were used for prediction. SVM and RF were employed for analysis, with SVM achieving an accuracy of 93% in predicting plants’ water content.

SVM has a strong advantage in handling nonlinear data and is widely used in agricultural research due to its ability to manage complex patterns. The data collected from RGB or hyperspectral images are high-dimensional and intricate, making SVM highly effective for classifying such data. SVM is particularly well suited for capturing subtle variations across different wavelength bands and, when combined with feature selection techniques, can produce more precise results. Additionally, SVM is adept at managing nonlinearity and high-dimensional data, making it superior in handling data complexity compared to other analysis methods. When analyzing data at various scales, such as with wavelet transforms, SVM excels at extracting the core patterns by combining time and frequency information. The following Table 9 provides a detailed analysis of the collected cases that utilized the SVM model.

### 5.2. PLS

Wan-Gyu et al. [54] proposed a method for detecting drought stress in soybeans using RGB images. Various vegetation indices were extracted from the RGB images to analyze changes in the leaf color and canopy cover of the soybean crops. PLS-DA was used as the analysis technique. The results indicated that leaf color changes were more sensitive to drought stress than canopy cover changes.

Sobejano-Paz et al. [103] proposed a method for assessing water stress in soybean and maize crops by combining hyperspectral and thermal imagery. Key variables included stomatal conductance, transpiration rate, and photosynthesis, with additional parameters such as plant temperature and canopy height included alongside hyperspectral data. PLS-R was used for the analysis, demonstrating accurate predictions for both soybeans and maize. For soybeans, temperature-related variables were identified as the primary factors, while for maize, canopy height was found to be the most significant variable.

Kang et al. [112] proposed a method for evaluating water stress in grapevines using hyperspectral imagery. Water stress was assessed by predicting leaf water potential, stomatal conductance, and soil moisture content. Spectral data were collected under diffuse lighting conditions. PLS-R was employed for analysis, demonstrating very high accuracy in predicting leaf water potential and soil moisture content.

The Partial Least Squares (PLS) algorithm effectively predicts key physiological variables such as stomatal conductance, transpiration rate, and photosynthesis. Specifically, the PLS-R model analyzes various wavelength bands to capture drought-sensitive spectra, enabling more precise predictions by utilizing wavelength bands that are not typically employed in conventional vegetation indices. Additionally, PLS excels in reducing the variability within spectral data, making it particularly strong in generating accurate predictions. The following Table 10 provides a detailed analysis of the collected cases that utilized the PLS model.

### 5.3. ANNs

Chandel et al. [57] proposed a method for detecting water stress in winter wheat crops using high-resolution thermal–RGB imagery combined with advanced AI techniques. The method integrated weather and soil data. LSTM was employed as the analysis technique, achieving a prediction accuracy of 96.7%.

Mazare et al. [61] proposed a thermal imaging analysis system for real-time detection of plant water stress. The system utilized a FLIR thermal camera to analyze plant temperature distribution and a deep learning algorithm was employed to learn and recognize early signs of water stress. An ANN was used as the analysis technique, achieving an accuracy of 97.8%.

Elsherbiny et al. [65] proposed a method for predicting water stress in rice crops using visible light and thermal imagery. Color and texture features were extracted from RGB images, while temperature indices were derived from thermal data. An ANN was employed as the analysis technique, utilizing a total of 21 key features as input variables. The model demonstrated a high accuracy of 99.4%.

Carrasco-Benavides et al. [66] proposed a method for predicting water stress in cherry trees using thermal imagery. Canopy temperature and relative humidity data were extracted from infrared thermal images. An ANN was used as the analysis technique to predict stem water potential and stomatal conductance, achieving accuracies of 83% and 75%, respectively. The model based on canopy temperature and stomatal conductance demonstrated an overall prediction accuracy of 81%.

King et al. [86] proposed a data-driven model for predicting the Crop Water Stress Index (CWSI) using canopy temperature in sugar beet and grape crops. A neural network model was employed for analysis, achieving a Nash–Sutcliffe efficiency greater than 0.88 in predicting the lower limit temperature (TLL) and an RMSE of less than 1.1 °C, indicating high accuracy.

Cherie et al. [87] presented a comparative study of AI techniques for calculating the Crop Water Stress Index (CWSI) in rice crops. Key meteorological variables such as relative humidity, air temperature, and canopy temperature were used to calculate the CWSI. FF-BP-ANN and SOM were employed as the analysis techniques, with FF-BP-ANN achieving the highest accuracy, at 97%.

Kumar et al. [91] proposed an AI-based method for predicting the Crop Water Stress Index (CWSI) in rice and Indian mustard crops. The model was compared with experimentally calculated CWSI values based on data collected under various irrigation levels. ANN, SVR, and ANFIS models were employed as the analysis techniques, with ANN5 (featuring five hidden neurons) achieving the highest accuracy, at 99%.

Muni et al. [95] presented a comparative study of AI techniques for predicting the Crop Water Stress Index (CWSI) in wheat crops. The CWSI values were derived from experimental data. MLP, SMOreg, M5P, RF, IBk, RT, bagging, and Kstar were used as the analysis techniques, with MLP showing the highest predictive accuracy, achieving an MAE value of 0.013.

Osco et al. [100] proposed a method for evaluating water stress in lettuce crops using hyperspectral imagery. Hyperspectral data were collected over 14 days from lettuce plants under induced stress conditions. An ANN was employed as the analysis technique, achieving an accuracy of 80% at the beginning of the experiment and 93% by the end.

Artificial Neural Networks (ANNs) excel at learning nonlinear relationships and handling multiple variables simultaneously, making them highly effective for accurately predicting complex issues such as crop water stress. Due to their hierarchical structure, ANNs can automatically learn key patterns in data, allowing for more refined predictions than other models. ANNs are particularly well suited for managing multiple physiological indicators, supporting precise water management and efficient irrigation decision-making. The following Table 11 provides a detailed analysis of the collected cases that utilized the ANN model.

### 5.4. CNNs

Zhuang et al. [53] proposed a method for detecting water stress in maize crops using phenotypic images of leaves. A total of 18,040 images reflecting three different water stress conditions were utilized. A CNN was employed as the analysis technique to extract feature maps, which were then used by an SVM classifier to categorize the water stress levels. The method achieved an accuracy of 88.41%.

Chandel et al. [56] proposed a system for real-time detection of crop water stress using an AI-based mobile device. GoogLeNet was employed to collect images in real time and classify water stress levels. The system achieved high accuracy rates of 97.9% for maize and 92.9% for wheat crops; additionally, it demonstrated fast processing speeds, with results generated within 200 milliseconds after image input.

Chandel et al. [57] proposed a method for detecting water stress in winter wheat crops using high-resolution thermal–RGB imagery combined with advanced AI techniques. The collected images were analyzed using the ResNet50 model, achieving high accuracy rates of 98.4% for thermal images and 96.9% for RGB images.

Melo et al. [67] proposed a method for detecting water stress in sugarcane crops using thermal imagery. Thermal images of sugarcane were collected with a thermal camera and analyzed using the Inception-ResNet-v2 model, which achieved 23% higher accuracy compared to manual evaluation. Specifically, the model attained accuracy rates of 83%, 90%, and 98% when predicting available water capacity (AWC) levels of 25%, 50%, and 100%, respectively, in sugarcane.

Aversano et al. [68] proposed a method for detecting water stress in tomato crops using thermal and optical imagery captured by drones. The VGG-19 model was employed for analysis, achieving an accuracy of 80.5% with the thermal images.

Jin et al. [70] proposed a method for detecting water stress in cotton crops under film-mulching drip irrigation using thermal imagery. The MobileNetV3 model was employed for analysis, achieving high accuracy, with an F1 score of 0.9990 and a processing speed of 44.85 ms.

Nasir et al. [102] proposed a method for estimating plants’ leaf water content using hyperspectral imagery. A CNN was employed for analysis, achieving a high accuracy of 98.4% and an RMSE of 4.183.

Sankararao et al. [104] proposed a method for detecting water stress in chickpeas using UAV-based hyperspectral imagery. The analysis was conducted using a 3D-2D CNN model, achieving a high accuracy of 95.44%.

CNN-based deep learning models demonstrate excellent performance in detecting water stress. CNNs can learn the phenotypic features of crops, allowing for non-invasive monitoring of water status and providing quantitative assessments of the degree of water stress. Models such as GoogLeNet and ResNet50 offer higher accuracy compared to other techniques, with prediction performance significantly improving through the integration of thermal imagery and multiple variables. Models such as DL-LSTM combine meteorological and soil variables to aid in real-time water management decisions, while transfer learning helps address data scarcity issues. MobileNetV3, with its fast processing speed and low computational complexity, is considered to be well-suited for agricultural applications, and 3D-2D CNN models accurately capture subtle stress variations by utilizing multiple bands. The following Table 12 provides a detailed analysis of the collected cases that utilized the CNN model.

### 5.5. Ensemble

Das et al. [63] proposed a method for detecting water status in vineyards using mobile thermal imaging and machine learning techniques. Random Forest (RF) was employed for analysis, achieving an R^2^ of 0.61 in cross-validation and 0.65 in predictions.

Yang et al. [64] proposed a method for detecting water stress in Chinese cabbage by predicting canopy temperature. Random Forest (RF) was used as the analysis technique, achieving high accuracy, with R^2^ values of 0.90 in the first experiment and 0.91 in the second experiment.

Wu et al. [69] proposed a method for estimating water stress in rice crops using multi-temporal temperature indices and machine learning techniques. Random Forest was employed for analysis, achieving R^2^ values of 0.78 for PWC, 0.77 for CWC, and 0.64 for CEWT, demonstrating high accuracy.

Wang et al. [71] proposed a method for detecting water stress in winter wheat crops using UAV-based multispectral and thermal remote sensing. The Gradient-Boosting Decision Tree (GBDT) technique was employed for analysis, achieving high accuracy, with R² values of 0.88 for NGS prediction and 0.90 for EWC prediction.

Katimbo et al. [89] proposed an AI-based model for predicting evapotranspiration (ET) and crop water stress. CatBoost and Stacked Regression were employed as the analysis techniques, achieving high accuracy, with an RMSE of 0.06–0.09 for CWSI prediction and 0.27–0.72 mm/day for ETc prediction.

Pei et al. [90] proposed a method for detecting water stress in cotton crops using UAV-based multispectral imagery and texture information. XGBoost was employed for analysis, achieving high accuracy, with an R^2^ of 0.90 and an RMSE of 0.05 for CWSI prediction.

Chen et al. [92] proposed a method for detecting water stress in sorghum and maize crops using UAV remote sensing and a multidimensional drought index. Random Forest Regression (RFR) was employed for analysis, achieving high accuracy, with R^2^ = 0.76 and RMSE = 1.15% for sorghum and maize.

Kapari et al. [94] proposed a method for detecting water stress in maize crops using multispectral and thermal images collected by UAVs, along with machine learning algorithms. Random Forest was employed for analysis, achieving high accuracy, with an R^2^ of 0.85 and an RMSE of 0.05 for CWSI prediction.

Loggenberg et al. [99] proposed a method for detecting water stress in grape crops using hyperspectral imaging and machine learning. Random Forest and XGBoost were employed as the analysis techniques, achieving accuracies of 83.3% and 80.0%, respectively.

Martin et al. [105] proposed a method for detecting water stress in potato crops using hyperspectral imaging and machine learning algorithms. Random Forest and XGBoost were employed for analysis, with XGBoost showing the highest performance across all growth stages, ultimately achieving an accuracy of 99.7%.

Niu et al. [106] proposed a method for predicting water stress in maize crops using UAV-based multispectral imagery. Random Forest, Artificial Neural Networks (ANNs), and Multivariate Linear Regression (MLR) were employed as analysis techniques, with the Random Forest model achieving the highest accuracy, recording an R^2^ of 0.89 and an RMSE of 0.066.

Thapa et al. [109] proposed a method for detecting water stress in grape crops using hyperspectral imagery and machine learning. Random Forest and Artificial Neural Networks (ANNs) were employed as analysis techniques, achieving 73% and 70% accuracy, respectively.

Mertens et al. [111] proposed a method for detecting water stress in maize using near-range thermal and hyperspectral imagery on an indoor automated plant phenotyping platform. Random Forest and LASSO were employed as analysis techniques, with the LASSO model achieving the highest accuracy, recording an R² of 0.63 and an RMSE of 0.47.

Mao et al. [114] proposed a method for predicting yield loss due to water stress in wheat crops using hyperspectral imagery. Various analysis techniques were employed, including Random Forest Regression (RFR), Partial Least Squares Regression (PLS-R), and multiple random ensembles. Among these, the multiple random ensemble model based on PLS-R demonstrated the highest accuracy.

Zhang et al. [116] proposed a method for predicting leaf water content (LWC) in rice using hyperspectral remote sensing combined with machine learning. The Gradient-Boosting Decision Tree (GBDT) technique was employed for analysis, achieving high accuracy, with R^2^ = 0.86 and RMSE = 0.01.

RF and GBDT have demonstrated strong performance in predicting the CWSI and crop water status, significantly improving accuracy by integrating meteorological and thermal data. CatBoost and XGBoost excel particularly in combining multispectral indices and texture information, making them crucial tools for real-time monitoring and irrigation management. LASSO has shown high accuracy in predicting evapotranspiration rates, and models utilizing spectral bands offer promising potential as efficient tools for water stress detection and management. The following Table 13 provides a detailed analysis of the collected cases that utilized the Ensemble model.

### 5.6. Others

Elsherbiny et al. [55] proposed a hybrid deep learning network for diagnosing the water status of wheat crops using IoT-based multimodal data. The analysis employed a hybrid model combining a CNN and LSTM, achieving an accuracy of 100%.

Das et al. [63] proposed a method for detecting water stress in wheat crops using UAV-based thermal imaging and machine learning. The analysis employed the Classification and Regression Tree (CRT) technique. The model achieved high accuracy, with an R² of 0.86 and RMSE of 41.3 g/m^2^ for biomass prediction, and an R^2^ of 0.78 and RMSE of 16.7 g/m^2^ for grain yield prediction.

Ismail et al. [32] proposed a method for smart agriculture that involves generating reconstructed thermal images from visible-light images. The analysis employed Generative Adversarial Networks (GANs) as the technique. The reconstructed thermal images demonstrated high accuracy and achieved visual quality comparable to actual thermal images.

Pradawet et al. [88] proposed a method for detecting water stress in maize crops using thermal imaging and machine learning models. They introduced an enhanced Crop Water Stress Index (CWSI), which demonstrated a high correlation with leaf stomatal conductance, achieving an R^2^ value of 0.90.

Bounoua et al. [93] proposed a method for predicting crop water stress using satellite-based remote sensing data. They employed CNN-LSTM and ConvLSTM models for analysis, with the CNN-LSTM model achieving the highest accuracy, with an RMSE of 0.119.

Asaari et al. [101] proposed a Convolutional Neural Network (CNN)-based regression method for detecting drought stress in maize crops and analyzing the recovery process using hyperspectral imaging. They combined Support Vector Machine (SVM) with K-Means Clustering to remove nonlinear effects and measure the spectral similarity of plants. The SVM classification achieved an accuracy of over 96%.

Adduru et al. [110] proposed a method for the early detection of water stress in peanut crops using UAV-based hyperspectral imaging and machine learning techniques. The analysis employed SVM, Random Forest (RF), and XGBoost. The SVM model achieved the highest accuracy, at 96.46%.

Malounas et al. [115] proposed a method for detecting drought stress in broccoli crops using hyperspectral imaging and AutoML (Automated Machine Learning). The analysis utilized PyCaret, AutoML, and PLS-DA (Partial Least Squares Discriminant Analysis). PyCaret achieved the highest accuracy, with an F1 score of 1.00.

In crop water stress research, which often involves extended study durations, the amount of available data can be limited. Therefore, Generative Adversarial Networks (GANs) hold significant potential for enhancing the accuracy of water stress detection across diverse environmental conditions through data augmentation. This capability positions GANs as a crucial tool for advancing precision agriculture. The following Table 14 provides a detailed analysis of the collected cases that utilized the different model.

## 6. Latest AI Technologies

### 6.1. Generative Adversarial Networks

**Figure 10:**
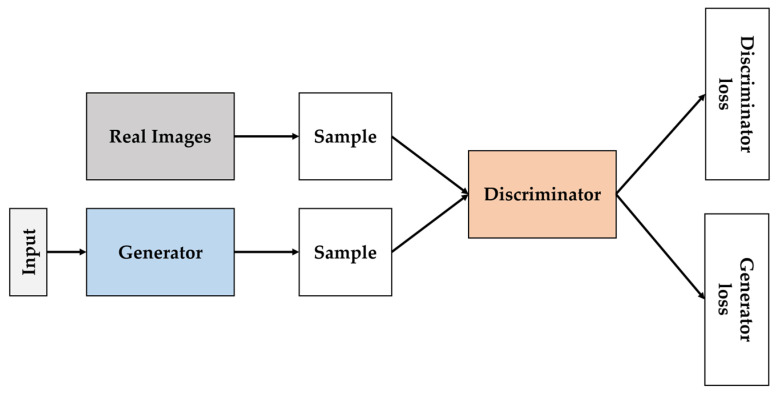
The basic structure of GAN. The generator creates fake images, and the discriminator learns to distinguish them from real ones, leading to increasingly realistic images over time.

To address the lack of data, researchers have used several techniques (Figure 10). The first is to augment the data by applying various geometric and color transformations, angular rotations, mirroring, etc., to the image [223,224,225,226,227,228,229]. The second way is to use transfer learning to improve performance [230,231,232,233,234,235,236]. These data augmentation techniques have led to improved model performance. In recent times, GANs [237] have gained traction as a third way to address data sparsity. GANs are a new generative modeling framework proposed by Goodfellow et al. that aims to generate data with the same characteristics as the training instances by synthesizing new data that are visually similar to the data in the training set [237]. Compared to traditional data augmentation techniques, GANs induce more variation and enrich the original dataset through representation learning, and they consistently improve model performance when combined with traditional augmentation methods and original data [238,239,240]. Various GAN architectures have been developed for image synthesis, including AutoGAN [241], BIGGAN [242], and DCGAN [243]. However, GAN models run the risk of producing low-quality images due to instability in training, and their performance is sensitive to hyperparameter settings [237,244,245]. Therefore, effective use of GANs requires careful hyperparameter tuning, network structure engineering, and various training tricks [246]. GAN operates by having the generator take random input to create fake images, while the discriminator compares the generated fake images with real images to distinguish between them. During the training process, the discriminator learns to better differentiate between real and fake images, and the generator improves its ability to create increasingly realistic fake images. Through this adversarial learning, the generator eventually produces refined images that can deceive the discriminator, making the generated images almost indistinguishable from real ones [247].

Research on crop water stress assessment using GANs has been collected in studies that propose methods to generate thermal images based on visible images [32]. Although there is a limited amount of prior research on crop water stress assessment using GANs, several works related to this topic have been carried out, including Data augmentation [248,249,250], disease detection [251], weed control [252], fruit detection [253], crop phenotyping [254], and quality assessment [255]. Considering these studies, GANs have great development potential in crop water stress assessment. Given the manual effort required, such as the tuning of hyperparameters, we believe that there is significant potential for further research in crop moisture stress assessment.

### 6.2. Explainable AI

**Figure 11:**
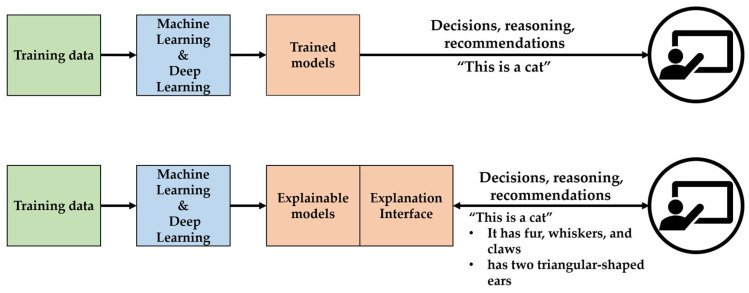
Comparison between general Machine Learning and Explainable AI (XAI) Approaches. The top section depicts the standard machine learning process, where models make decisions without explanations. In contrast, the bottom section shows the XAI approach, which generates explainable models and provides transparency through an explanation interface.

Explainable AI (XAI) is a technology that enables end users to understand the learning models and decision-making processes of AI systems, helping them to trust AI systems [256]. The main goals of XAI are to build user trust, increase transparency and accountability, and improve model performance by making it possible to understand how AI models work and how they make decisions [257,258]. In recent years, XAI has rapidly gained popularity, with new interpretable machine learning methods being proposed, reviewed, and applied in various scientific fields [259,260,261,262]. XAI enhances model transparency, helps users trust the system, supports the decision-making process, and makes it easier to debug and improve models [263]. However, there are many limitations, including difficulties in interpreting complex models, variability in posterior models, data bias, and misunderstanding of causality. To overcome these limitations, using hybrid models seems to be an effective way to simplify the model and balance performance [264] (Figure 11).

Crop water stress assessments rely on various parameters (soil moisture content, canopy temperature, photosynthesis rate, chlorophyll content, etc.). XAI can help analyze and interpret these data and clarify how each parameter contributes to the assessment of water stress. However, there is still a lack of published research using XAI to assess water stress in crops. However, XAI is already being used in a variety of agricultural applications, including crop recommendations [258], agricultural data analytics [265], and yield prediction [266], and it is beneficial in detecting diseases in crops [267,268,269]. Since water stress in crops can be considered a component of disease, the applicability of XAI technology to crop water stress assessment is considered to be high. Considering the complex model interpretation, it is expected that research on crop water stress assessment using XAI will be actively conducted in the future.

## 7. Conclusions

The assessment of crop water stress is a crucial process for agricultural productivity and resource management. To conduct this assessment effectively, utilizing remote sensing technologies is essential. This study provides a comprehensive analysis of remote sensing and artificial intelligence (AI) techniques for the non-destructive and efficient evaluation of crop water stress induced by climate change. The main conclusions are as follows:

- The use of remote sensing technologies has demonstrated the potential for non-destructive and precise evaluation of crop water stress. In particular, the use of thermal imaging data has proven effective, and CWSI-based thermal analysis holds significant potential for rapid and accurate water stress assessment.
- Data analysis utilizing machine learning and deep learning models shows high potential for predicting crop water stress. Notably, CNN-based models are expected to achieve excellent performance through RGB and thermal imaging data.
- Ensemble learning techniques combining various models have shown superior prediction performance compared to single models. Ensemble models such as RF and XGBoost can effectively learn complex data patterns and contribute to improved prediction accuracy.
- The research on generating thermal images based on visible-light images using GANs has a high potential for addressing data scarcity issues. Reconstructed thermal images are expected to effectively assess water stress conditions.
- Explainable AI (XAI) contributes to increasing user trust by explaining the decision-making processes of AI models. XAI is useful in interpreting the impact of various variables in water stress assessment and holds promise for future applications.

Overall, this study has derived high-accuracy models. However, developing technologies that can adapt to accelerating climate change and variable production conditions remains a crucial challenge. The application and development of various AI techniques are necessary to overcome the limitations of existing models. In particular, crop research typically takes about one year, and any management errors could prevent achieving the desired results within that year. Upon analyzing the collected cases, most studies either built models with a small number of images or increased data through automatic capture every five to ten min. However, this only increases the quantity of data, not its quality. Generative algorithms like GANs are expected to make a significant contribution to addressing this data scarcity issue. Additionally, while XAI algorithms have not yet been directly applied to water stress evaluation, they could be useful in considering complex variables by improving model transparency. This study is anticipated to contribute to solving data scarcity issues and enhancing the accuracy and efficiency of decision-making in future crop water stress assessments.

## Figures and Tables

**Table 1:** Search term strings per database. A comprehensive review of the literature was conducted to analyze the application of machine learning and deep learning techniques in evaluating crop water stress using different data modalities. The total number of collected research papers for each modality was as follows: for Crop Water Stress Index (CWSI), 21 papers were identified; for RGB images, 11 papers were collected; for thermal images, 32 papers were gathered; and for hyperspectral images, 31 papers were found.

Keyword	Search Terms and Criteria	Number of Papers
CWSI	Machine learning, Deep learning, Water stress, Crop, CWSI	21
RGB	Machine learning, Deep learning, Water stress, Crop, RGB	11
Thermal	Machine learning, Deep learning, Water stress, Crop, Thermal	32
Hyperspectral imagery	Machine learning, Deep learning, Water stress, Crop, Hyperspectral	31

**Table 2:** RGB imaging for crop water stress evaluation.

Crop	Best Model	Methodologies	Objective	Authors	Publisher	Nation	Year
Chickpea	SVM	SVM, K-Nearest Neighbors, DT, Naive Bayes (NB), Discriminant Analysis (DA)	Using images of chickpea shoots to identify crop water stress due to low soil moisture	[52]	IEEE	India	2020
Maize	Convolutional Neural Network (CNN)	CNN	Recognizing and quantifying water stress in maize using digital imagery	[53]	Elsevier	China	2020
Soybean	Partial Least Squares Discriminant Analysis (PLS-DA)	Partial Least Squares Discriminant Analysis	Applicability and limitations of RGB image-based crop vigor indices in determining chilling stress in soya beans	[54]	Korean Society of Agrometeorology	Korea	2021
Wheat	CNN-LSTM-CNN	CNN, Long Short-Term Memory (LSTM), CNN-CNN, LSTM-LSTM, CNN-LSTM-CNN	Identification and automatic detection of water stress in wheat crops	[55]	Elsevier	China	2022
Wheat and maize	GoogLeNet	AlexNet, GoogLeNet, Inception V3, MobileNet V2, ResNet-50	Development of a device for real-time assessment of water stress in wheat and maize crops	[56]	Elsevier	India	2024

**Table 3:** Thermal imaging for crop water stress evaluation.

Crop	Best Model	Methodologies	Objective	Authors	Publisher	Nation	Year
-	ANN	ANN	Implementing a system for monitoring water stress in crops	[61]	IEEE	Romania	2018
Grapes	-	Rotation Forests (ROF), DT	Thermal-image-based estimation and field assessment of water stress in grapes	[62]	PLOS	Spain	2018
Wheat	Classification and Regression Tree (CRT)	CRT algorithm	Thermal-image-based biomass and grain yield prediction of wheat grown under moisture stress in sodic soil environments	[63]	Elsevier	Australia	2021
Brassica	Random Forest (RF)	RF	Assessing crop moisture status with simulated baseline canopy temperature and predicted CWSI for brassica in China	[64]	MDPI	China	2021
Rice	ANN	ANN	Canopy moisture content prediction based on thermal–RGB imaging in rice	[65]	MDPI	China	2021
Cherry	ANN	ANN	Thermal-image-based assessment of cherry moisture status	[66]	Elsevier	Chile	2022
Sugarcane	Inception-Resnet-v2	Inception-Resnet-v2	Predicting water stress in sugarcane crops based on thermal imagery	[67]	Elsevier	Brazil	2022
Tomato	VGG-19	VGG-19	Water stress classification in tomato crops based on thermal and optical aerial imagery	[68]	J.UCS	Italy	2022
Wheat	ResNet50	ANN, K-Nearest Neighbors (KNN), Logistic Regression (LO), SVM, LSTM	Water stress assessment using thermal–RGB imaging in winter wheat	[57]	MDPI	India	2022
Rice	Generative Adversarial Network (GAN)	GAN	Monitoring moisture stress with reconstructed thermal images	[32]	IEEE	Indonesia	2022
Rice	RF	RF	Moisture-parameter-based moisture status estimation in rice using thermal imagery	[69]	Elsevier	China	2023
Cotton	MobilenetV3	VGG16, ResNet-18, MobilenetV3, DenseNet-201, CSPdarknet53	Predicting water stress in cotton crops based on thermal imagery	[70]	Elsevier	China	2024
Wheat	Gradient-Boosting Decision Tree (GBDT)	GBTD, PLS, SVM	Diagnosing water stress in wheat growth	[71]	Elsevier	China	2024

**Table 4:** CWSI for crop water stress evaluation.

Crop	Best Model	Methodologies	Objective	Authors	Publisher	Nation	Year
Sugar beet, wine grape	Nash–Sutcliffe	Nash–Sutcliffe, linear model	Estimating baseline canopy temperature for CWSI calculations	[86]	ASABE	USA	2020
Rice	FF-BP-ANN	Self-Organizing Maps (SOM), Feedforward Backpropagation Artificial Neural Network (FF-BP-ANN)	Using machine learning techniques to determine optimal CWSI values for rice	[87]	Taylor & Francis	India	2023
Maize	Linear regression (LR)	LR	Development of a thermal-imaging-based CWSI approach for the assessment of water stress and yield prediction in maize	[88]	Wiley	Thailand	2023
Maize	CatBoost	ANN, LSTM, RF, CatBoost, SVM, KNN, Multiple Linear Regression (MLP), Stacked-RF, Stacked Regression, Weighted Ensemble	CWSI prediction for corn crops	[89]	Elsevier	USA	2023
Cotton	Extreme Gradient Boosting (XGBoost)	SVM, XGBoost, Backpropagation Neural Network (BPNN)	Evaluation of CWSI estimation during the cotton growing season based on UAV multispectral imagery	[90]	Elsevier	China	2024
Wheat, mustard	ANN5 (ANN with five hidden neurons)	SVM, ANN, Adaptive Neuro-Fuzzy Inference System	CWSI prediction using relative humidity, air temperature, and canopy temperature	[91]	Research Square	India	2024
Sorghum, maize	RF	RF, SVM, PLS	Comparing the applicability of the CWSI to the Three-Dimensional Drought Index (TDDI), which consists of temperature, air temperature, and five vegetation indices.	[92]	Elsevier	China	2024
Citrus	Long sequences: CNN-LSTM; short sequences: ConvLSTM	ConvLSTM, CNN-LSTM	CWSI-based water stress prediction	[93]	MDPI	Morocco	2024
Maize	RF	PLS, SVM, RF	Determining water stress indices for monitoring and mapping crop water stress variability	[94]	MDPI	South Africa	2024
Wheat	MLP	MLP, SMOreg, M5P, RF, IBK, Random Trees (RT), bagging, Kstar	CWSI prediction for wheat crops	[95]	ASCE	India	2024

**Table 5:** Multispectral and hyperspectral imaging for crop water stress evaluation.

Crop	Best Model	Methodologies	Objective	Authors	Publisher	Nation	Year
Grapes	RF	XGBoost, RF	Hyperspectral-data-based water stress assessment in grapes	[99]	MDPI	South Africa	2018
Lettuce	ANN	ANN	Hyperspectral-data-based water stress assessment in lettuce	[100]	MDPI	Brazil	2019
Maize	SVM and K-means Clustering Algorithm	SVM and K-Means Clustering Algorithm	Hyperspectral-data-based analysis for water stress assessment and recovery in maize	[101]	Elsevier	Belgium	2019
A variety of leaves	CNN	CNN	Estimating leaf water content to quantify water stress	[102]	IEEE	Pakistan	2019
Soybeans, maize	PLSR	PLSR	Assessing plants’ physiological water stress responses	[103]	MDPI	Denmark	2020
Chickpeas	3D to 2D CNN	3D to 2D CNN	Assessing water stress in chickpeas based on hyperspectral data acquired by UAVs	[104]	IEEE	India	2021
Potatoes	RF, XGBoost	RF, MLP, CNN, SVM, XGBoost, AdaBoost	Hyperspectral-data-based water stress assessment in potatoes	[105]	MDPI	Colombia	2021
Maize	RF	RF, ANN, MLR	Managing water stress in maize crops and estimating crop traits	[106]	Elsevier	China	2021
Maize	SVM	RF, SVM	Moisture stress detection and optimal wavelength region selection based on hyperspectral data during corn’s grain-filling stage	[107]	IEEE	India	2022
Pearl millet	RFE-SVM	SelectFromModel RF (SFM-RF), SelectFromModel SVM (SFM-SVM), SelectFromEnsemble RF (SFE-RF), Recursive Feature Elimination SVM (RFE-SVM), Chi2	Identifying canopy moisture stress in pearl millet crops	[108]	IEEE	India	2022
Grapes	RFC	Optimized RF Classifier (RFC), ANN	Hyperspectral-data-based water stress assessment in grapes	[109]	ASABE	USA.	2022
Peanuts	-	SelectFromModel RF (SFM-RF), SelectFromModel SVM (SFM-SVM), SelectFromEnsemble RF (SFE-RF), Recursive Feature Elimination SVM (RFE-SVM)	Canopy water stress assessment based on hyperspectral data in peanuts	[110]	IEEE	India	2023
Maize	RF	LASSO, PLSR, RF	Monitoring plant water stress for plant transpiration rates	[111]	SpringerLink	Belgium	2023
Grapes	PLS	PLS	Soil moisture and grape water stress detection based on hyperspectral data under diffuse illumination	[112]	Elsevier	USA	2023
Wheat	SVM	Wavelet Index Model, MLR, RF, SVM	Monitoring moisture status in winter wheat	[113]	SpringerLink	China	2023
Wheat	(multi-random ensemble on PLSR) MRE-PLSR	RFR (RF Regression), PLSR, MRE-PLSR	Predicting yield at different growth stages of wheat crops under moisture stress conditions	[114]	Elsevier	China	2024
Broccoli	PyCaret	PyCaret, PLS-DA	Assessment of water stress in broccoli based on AutoML and hyperspectral data	[115]	Elsevier	Greece	2024
Rice	GBDT	GBDT	Integrating leaf moisture data from multiple rice varieties to create a model to estimate crop moisture status	[116]	SpringerLink	China	2024

**Table 6:** Examples of using machine learning to analyze crop water stress.

Algorithms Used	Number Uses	Percentage (%)
SVM(R)	6	23.6%
PLS(DA)	3	11.5%
KNN	2	7.7%
DT	2	7.7%
SVM-based models	2	7.7%
DA	1	3.8%
NB	1	3.8%
ROF	1	3.8%
K-Means	1	3.8%
CRT	1	3.8%
LO	1	3.8%
RFC	1	3.8%
PyCaret	1	3.8%
SOM	1	3.8%
LR	1	3.8%
ANFIS	1	3.8%
Total	26	100%

**Table 7:** Examples of using deep learning to analyze crop water stress.

Algorithms Used	Number Uses	Percentage (%)
CNN-based models	9	47.3%
ANN-based models	6	31.6%
BPNN	2	10.5%
MLP	1	5.3%
LSTM	1	5.3%
Total	19	100%

**Table 8:** Examples of using ensemble learning to analyze crop water stress.

Algorithms Used	Number of Uses	Percentage (%)
RF	5	38.4%
XGBoost	3	23.1%
RF-based models	2	15.4%
SVM and K-means Clustering Algorithm	1	7.7%
Inception-Resnet-v2	1	7.7%
AdaBoost	1	7.7%
Total	13	100%

**Table 9:** Case analysis with SVM.

Authors	Study Area	Study Period	Data Acquisition Methods	Number of Data	Accuracy
[52]	National Institute of Plant Genome Research (NIPGR)	5 months	The images were taken indoors using a Canon camera in auto mode.	A total of 8000 images were collected, with 240 images per plant.	73%
[107]	Institute of Agricultural Research, Chinese Academy of Agricultural Sciences	From October 2020 to May 2022	Measurements were taken directly in the field using a handheld spectrometer.	A total of 246 sample data points.	93%
[108]	Xinxiang City in Henan Province and Xingtai City in Hebei Province, China.	From October 2022 to June 2023	Hyperspectral data was collected during flight using a drone.	A total of 900 samples were collected from 12 treatments in Xinxiang and Xingtai, with 30 samples per treatment in Xinxiang and 45 in Xingtai.	95.38%
[113]	Henan Province, China	2020 to 2022	-	-	93%

**Table 10:** Case analysis with PLS.

Authors	Study Area	Study Period	Data Acquisition Methods	Number of Data	Accuracy
[54]	Central Institute of Agricultural Engineering located in Bhopal, India	from July to October 2020	RGB images were captured from the top of the rainout shelter using a commercial digital camera.	RGB images were captured a total of 26 times.	-
[103]	Riso Environmental Risk Assessment Facility (RERAF) in Roskilde, Denmark	from March to June 2018	Data were collected directly using a FLIR Tau2 324 camera (thermal imaging) and a Cubert UHD 185 camera.	144 soybean samples, 126 maize samples	92%
[112]	a *Vitis vinifera* L. cv. Riesling vineyard located in Prosser, Washington, USA	In 2021	Spectral data were collected using a ground-based hyperspectral camera.	A total of 179 leaf samples and 62 soil moisture samples	89%

**Table 11:** Case analysis with ANN.

Authors	Study Area	Study Period	Data Acquisition Methods	Number of Data	Accuracy
[57]	Research farm at the Central Institute of Agricultural Engineering (CIAE) located in Bhopal, India.	From 2019 to 2021.	Images were collected from a distance of 1 m using an integrated thermal-RGB imaging system based on Raspberry Pi.	A total of 3200 images (1600 RGB and 1600 thermal images).	96.7%
[61]	University of Pitesti and Polytechnic of Bucharest, Romania	In 2018	Images were automatically captured every 1 to 10 min using a FLIR thermal camera (300 × 128 resolution).	A total of 50,000 images	97.8%
[65]	Zhejiang University located in Hangzhou, Zhejiang, China	From 10 July to 21 September 2018	Captured directly using a Canon PowerShot SX720 HS camera and a FLIR Tau2-640 thermal imaging camera.	A total of 400 images were captured, of which 360 images were used.	99.4%
[66]	A cherry orchard spanning 13.2 hectares in the Curicó region of Chile.	2017–2018 and 2018–2019.	Captured at a distance of 3.5 m using a FLIR thermal imaging camera (TIS60, Fluke Corporation).	Collected physical indicators and thermal imaging data from a total of 24 trees.	83%
[86]	Idaho, Wyoming, and Oregon in the United States.	Over a period of five years.	Collected based on directly measured canopy temperature and surrounding environmental data.	-	88%
[87]	Irrigation Laboratory at the Indian Institute of Technology, Roorkee.	During the rice growing season.	Data collected from laboratory measurements of meteorological variables: relative humidity, air temperature, and canopy temperature.	-	97%
[91]	Agricultural research station at the National Institute of Technology in Hamirpur, India.	From 2017 to 2019.	Humidity and air temperature were recorded every 10 min, and canopy temperature was measured with a portable infrared thermometer.	Indian mustard: 1260 for development, 1350 for validation; wheat: 1530 for development, 1458 for validation.	99%
[95]	-	From December 2022 to April 2023.	Data were directly collected using a portable infrared radiometer and a weather observation station.	-	-
[100]	Growth chamber (Phytotron) under controlled conditions at the Federal University of Mato Grosso do Sul in Brazil.	-	Spectrum data were collected in the range of 325–1075 nm using a FieldSpec HandHeld ASD Spectroradiometer.	360 spectral signatures were measured, with 90 collected over four days of the experiment.	93%

**Table 12:** Case analysis with CNN.

Authors	Study Area	Study Period	Data Acquisition Methods	Number of Data	Accuracy
[53]	Shaanxi Province, China.	The experiment began on 18 June 2014 and continued throughout the plant growth period.	Images were collected from a height of 4.5 m using a CCD camera mounted on a fixed platform.	A total of 18,040 digital images	88.41%
[56]	Central region of India.	From November 2021 to June 2022.	Image data were collected in the field using a Raspberry Pi device and various RGB cameras, including a Canon PowerShot SX740, Raspberry Pi camera, and smartphone.	A total of 3200 RGB images	97.9% for maize and 92.9% for wheat
[57]	Research farm at the Central Institute of Agricultural Engineering (CIAE) located in Bhopal, India.	From 2019 to 2021.	Images were collected from a distance of 1 m using an integrated thermal-RGB imaging system based on Raspberry Pi.	A total of 3200 images (1600 RGB and 1600 thermal images).	98.4%
[67]	University of São Paulo (USP/ESALQ) located in São Paulo, Brazil.	From 11 August 2019, for a duration of 120 days.	Images were collected using a FLIR ONE Pro LT thermal imaging camera, which was connected to a smartphone.	A total of 4050 thermal images	-
[68]	A tomato farm near Benevento, southern Italy.	The entire growth cycle of tomato crops.	Collected thermal and optical images using a drone (UAV).	6600 thermal and 6600 optical images	80.5%
[70]	Shihezi University in the Xinjiang region of China.	From May 2023 to August 2023, a total of 150 days.	The FLIR ONE Pro thermal imaging camera was connected to a smartphone for use.	A total of 1300 thermal images	-
[102]	Institute of Space Technology located in Islamabad, Pakistan.	-	The reflection spectra of plant leaves were collected in the laboratory using a spectroradiometer.	402 image data sets were collected for 11 plant species, with each image containing 3457 spectral bands.	98.4%
[104]	International Crops Research Institute for the Semi-Arid Tropics (ICRISAT) located in Hyderabad, India.	-	Data were collected using a Pika-L Hyperspectral Imaging (HSI) camera mounted on a drone (UAV).	208 data lines were collected, with 26 genomes per treatment and 8 repetitions.	95.44%

**Table 13:** Case analysis with Ensemble.

Authors	Study Area	Study Period	Data Acquisition Methods	Number of Data	Accuracy
[63]	Conducted at two locations in the Goondiwindi region of Australia: medium salinity soil (MS) and high salinity soil (HS).	From May to November 2018.	Thermal images were collected with a FLIR Tau 2 camera on a DJI Matrice 600 Pro drone, along with a MicaSense RedEdge-M multispectral camera.	-	-
[64]	South China Agricultural University located in Guangzhou, China.	From 27 November 2020 to 31 December 2020, and from 25 May 2021 to 20 June 2021.	Canopy temperature was measured at 0.3 m every 10 min with an infrared radiometer, while weather sensors recorded air temperature, humidity, wind speed, and photosynthetically active radiation.	-	0.91%
[69]	Luogao Experimental Base in Jiangsu Province, China.	From 2019 to 2020.	Thermal images were collected using a FLIR SC620 thermal camera from a height of 1 m, at 2-hour intervals between 8 AM and 4 PM.	A total of 205 data	78%
[71]	China Agricultural University Experimental Station in Zhuozhou, Hebei Province, China	March to June, 2021 and 2022	UAV multispectral and thermal remote sensing	14 vegetation indices and 2 thermal indices measured over 6 key growth stages	90%
[89]	West Central Research, Extension, and Education Center, University of Nebraska-Lincoln, Nebraska, USA	2020 and 2021	Sensor data assimilation (weather sensors, soil moisture sensors, infrared thermometers, etc.), real-time climate and soil moisture monitoringh	540 total data points (30 days × 3 months × 2 years)	-
[90]	Yuli County, Xinjiang, China, in the alluvial plain downstream of the Tarim and Peacock Rivers	Cotton sown on 4 April 2021, and harvested on 20 September 2021	UAV-based multispectral and thermal imaging using MicaSense Altum camera attached to DJI M200 V2 UAV	2946 valid images collected over five field measurement dates	90%
[92]	Jichangbuyi Miao Township, Anshun City, Guizhou Province, China	Crops were sown in May 2023, with data collected until maturity in August.	A DJI Matrice300 RTK UAV equipped with MS600Pro multispectral and Zenmuse H20T thermal-infrared sensors was used.	Ground-based VMC data were collected from 155 samples (108 for training and 47 for validation).	76%
[94]	Smallholder farm in southern Africa, specifically in the Swayimana rural area, uMshwathi Local Municipality, KwaZulu-Natal Province, South Africa.	8 February 2021 to 26 May 2021.	Collected using a DJI Matrice 300 UAV equipped with a MicaSense Altum sensor and a handheld infrared thermometer.	3576 images	85%
[99]	Welgevallen experimental farm, Stellenbosch, Western Cape, South Africa.	-	Terrestrial hyperspectral imaging using the SIMERA HX MkII hyperspectral sensor.	A total of 60 leaf spectra samples	83.3%
[105]	Tibaitatá Research Center, Corporación Colombiana de Investigación Agropecuaria (AGROSAVIA), Cundinamarca, Colombia	In 2021	Hyperspectral imagery (400–1000 nm) using 128 spectral bands from a Surface Optics Corporation 710-VP camera	A total of 116 images	99.7%
[106]	A 1.13-hectare maize field located in Zhaojun Town, Inner Mongolia, China	2018 and 2019 growing seasons	UAV imagery was collected using a self-developed hexacopter equipped with a MicaSense RedEdge camera for multispectral imagery, and a DJI Phantom 4 Pro for RGB imagery.	A total of 165 multispectral and 161 RGB images were collected in 2018, and 135 multispectral and 134 RGB images in 2019.	89%
[109]	An experimental vineyard in arid southeastern Washington, USA.	Data collected over two growing seasons.	Hyperspectral images acquired from a ground-based utility vehicle.	-	73%
[111]	PHENOVISION automated phenotyping platform, a semi-controlled greenhouse, Belgium.	-	Proximal thermal and hyperspectral imaging using a high-throughput plant phenotyping platform.	14,744 images and 288 additional physiological trait images were collected.	63%
[114]	Comprehensive Experimental Base of the Chinese Academy of Agricultural Sciences, Xinxiang City, Henan Province and Yanli Experimental Base, Xingtai City, Hebei Province, China	From 2022 to 2023	UAV-based hyperspectral data acquisition using a DJM600 Pro UAV equipped with a Resonon Pika L nano-hyperspectral scanner.	-	-
[116]	Hefei, Anhui Province and Fuyang, Anhui Province, China	From 2021 to 2022	Hyperspectral remote sensing using the ASD FieldSpec 4 device for spectral data collection.	A total 91 sample data points	86%

**Table 14:** Case analysis using different models.

Authors	Study Area	Study Period	Data Acquisition Methods	Number of Data	Accuracy
[55]	Zhejiang University, Hangzhou, China	from October 2019 to January 2020.	IoT-based multimodal data acquisition, including RGB images, soil moisture sensors, air temperature, relative humidity, and wind speed.	876 images expanded to 5256 with augmentation.	100%
[63]	Goondiwindi, northeastern grains growing region, Australia.	2018 wheat growing season (May to November).	UAV thermal remote sensing with a FLIR Tau 2 camera on a DJI Matrice 600 Pro, collecting data	-	-
[32]	Ismail, Daddy Budiman, Ervan Asri, Zass Ressy Aidha	-	Visible images were used to generate thermal images using a deep learning-based GAN model.	-	-
[88]	Phitsanulok Province, Thailand (Thapo sub-district and Wang Thong district)	from 2018 to 2020	Data were collected using a FLIR C2 camera for thermal imaging positioned above the crop canopy, along with soil moisture sensors and weather data.	-	90%
[93]	Ourgha Farm, Khnichet rural commune, Sidi Kacem Province, Morocco	from 2015 to 2023	Remote sensing data obtained from Landsat 8 satellite images processed through Google Earth Engine, focusing on the Crop Water Stress Index (CWSI).	50 Landsat 8 satellite images	-
[101]	PHENOVISION high-throughput phenotyping platform, VIB, Ghent, Belgium	50 days of plant growth were monitored, beginning from the V2 growth stage.	Hyperspectral imaging using a VNIR-HS line scan push-broom camera (ImSpector V10E), capturing images across 194 spectral bands (400–1000 nm).	1900 hyperspectral images were collected from six drought treatment groups.	96%
[110]	International Crops Research Institute for the Semi-Arid Tropics (ICRISAT), Hyderabad, India	from November 2021 to February 2022.	Hyperspectral imaging using a Resonon Pika-L camera on a DJI Matrice-600 Pro UAV, capturing 300 bands (385–1020 nm).	16,000 samples were collected with 1000 per genotype per class.	96.46%
[115]	Agricultural University of Athens, Athens, Greece	In 2024	Hyperspectral imaging with a Snapscan VNIR camera on a three-wheel platform using natural sunlight.	120 images were captured, reduced to 90 after outlier removal (42 from drought onset, 48 from acclimation).	-
